# Cardiac Simulator Technologies and Design for Medical Education and Auscultation Training: A Systematic Review

**DOI:** 10.3390/bioengineering12070731

**Published:** 2025-07-03

**Authors:** Christian Romero-Martínez, Luis Adrián Zúñiga-Avilés, Giorgio M. Cruz-Martínez, José Javier Reyes-Lagos, Joel Zagoya-López, Ángel Eduardo Bárcenas-García

**Affiliations:** 1 School of Medicine, Autonomous University of Mexico State, Toluca 50180, Mexico; cromerom831@alumno.uaemex.mx (C.R.-M.); jjreyesl@uaemex.mx (J.J.R.-L.); jzagoyal@uaemex.mx (J.Z.-L.); aebarcenasg@uaemex.mx (Á.E.B.-G.); 2 Program Secihti-Faculty of Engineering, Autonomous University of Mexico State, Toluca 50130, Mexico; 3 School of Engineering, Autonomous University of Mexico State, Toluca 50180, Mexico; gmcruzm@uaemex.mx

**Keywords:** cardiac simulator, cardiac auscultation, medical training

## Abstract

Medical simulators have revolutionized clinical training, particularly in teaching skills such as cardiac auscultation. This review synthesizes recent advances in the technological design and implementation of cardiac simulators for medical education, alongside scientometric and patentometric analyses. The focus is on innovations enhancing efficacy, safety, and accessibility. Analyses included 69 patents published over the past five years, sourced from Google Patents, Patentscope, Espacenet, and The Lens. A bibliometric analysis was performed using 52 scientific reports from PubMed, ScienceDirect, and The Lens within the same timeframe. Key findings indicate an 8% increase in AI-integrated cardiac auscultation devices compared to conventional equipment. Furthermore, 85% of the studies reported compliance with applicable regulations of at least 90%, reflecting improved regulatory alignment. This analysis provides a foundation for future research and the development of more accurate and accessible educational tools for cardiac auscultation training.

## 1. Introduction

In recent decades, technological advances have revolutionized the field of cardiovascular medicine, driven mainly by three fundamental factors: the increase in the prevalence of heart disease, the need to improve diagnostic accuracy, and the growing demand for more effective educational tools in medical training. According to the World Health Organization (WHO), cardiovascular disease (CVD) is the leading cause of death worldwide. An estimated 17.9 million people died in 2019 because of CVD, representing 32% of all deaths worldwide [1].

In Mexico, this reality is no different. According to data from the National Institute of Statistics and Geography (INEGI), during the period of January–June 2022, deaths due to heart disease were the leading cause of death nationwide, with 105,864 cases [2]. Timely attention is essential to avoid serious, irreversible sequelae and even death due to cardiovascular diseases, since up to 90% of infarcted persons survive when treated in time, according to the National Institute of Cardiology “Ignacio Chávez” [3]. Timely treatment is the action that follows as soon as the disease is diagnosed. It is important to understand that treatment is most effective if it is started as quickly as possible [4].

**Figure 1:**
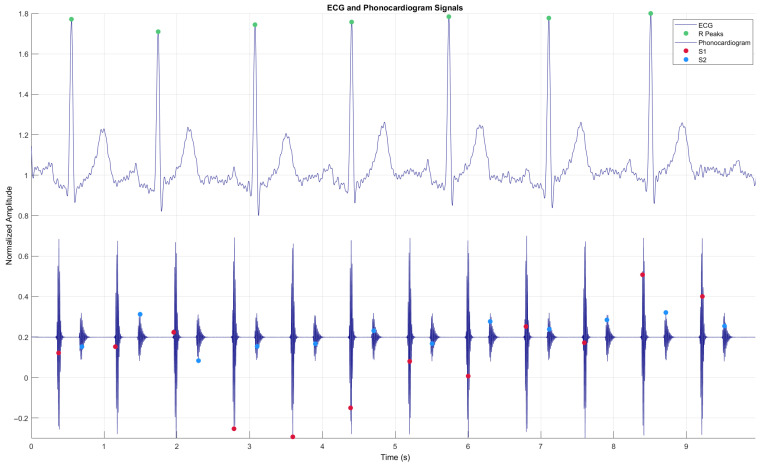
Simulated simultaneous signals from a phonocardiogram (PCG) and an electrocardiogram (ECG), including the R-peaks of the ECG, showing the four states of the PCG: S1—Systole, S2—Diastole.

In this context, cardiac auscultation continues to be a fundamental diagnostic tool in clinical practice, allowing early detection of various cardiovascular pathologies. However, mastering this skill poses a significant challenge in medical education, requiring extensive practice and exposure to a wide range of cardiac sounds, both normal and pathological. Traditional teaching of cardiac auscultation faces significant limitations, including reduced availability of patients with specific conditions, variability in the presentation of clinical signs, and difficulty in repeated exposure to heart sounds [5]. An electrocardiogram (ECG) is a signal derived from the electrical activity of the heart, while a phonocardiogram (PCG) is a recording of the sounds that are produced during its activity [6]. The combination of ECG and PCG leads to higher performance of the evaluation of the cardiac condition than a diagnosis based only on PCG recordings, according to results such as accuracy: 92.5% vs. 82.5%, AUC: 0.9505 vs. 0.9066, sensitivity: 92.31% vs. 76.92%, and specificity: 92.86% for both [7]. (see Figure 1). The first heart sound (S1) is produced when the atrioventricular valves (mitral and tricuspid) close at the beginning of systole, while the second heart sound (S2) is generated by the closure of the semilunar valves (aortic and pulmonary) after systole. R peaks refer to sharp upward deflections in an ECG signal, representing ventricular depolarization.

The applications of simulation in healthcare can be categorized into 11 dimensions: goals and purposes of the simulation activity; unit of participation; level of experience of participants; scope of healthcare; professional discipline of participants; type of knowledge, skills, attitudes, or behaviors addressed; age of the simulated patient; technology applicable or needed; location of simulation; degree of direct participation; and method of feedback used. Using simulation to improve safety will require the full integration of its applications into routine healthcare structures and practices [8]. One particular type of simulation is based on high-fidelity mannequins, which reproduce many of the characteristics of patients and make it possible to recreate highly realistic scenarios [9].

A simple classification of cardiac simulators is divided into those based on physical models, those that use computers to create illusions of reality, and those that combine the two models [10]. The initial step in these simulations is provided by mannequins that, in addition to anatomical reproduction, are equipped with software that reproduces the function of some organs and allow the student to apply their knowledge; the most obvious example is the equipment in which a thorax reproduces cardiac and pulmonary semiology with enough realism. The student can identify and distinguish between different options and recognize the syndrome to which they are linked. The second link is provided by complex interactive robotics, where a complete human body is replicated using software that equips the doll with all the cardiac, vascular, and pulmonary functions. This allows for the design of complete clinical syndromes or cases: the student must explore the robot, arrive at a clinical orientation, and initiate a set of basic skills if the situation requires it. From here, the level of complexity can be increased [11].

This work is organized into five sections that allow a clear and orderly understanding of the development of the study. Section 2 reviews the methodology based on some aspects suggested by the PRISMA framework. Section 3 presents the results obtained from the analysis of the data, presenting the information objectively by means of tables, graphs, and descriptions. Section 4 corresponds to the discussion, where the results are interpreted, highlighting relevant findings. Finally, Section 5 presents the conclusions, in which the main contributions of the research are synthesized.

## 2. Review Methodology

The search strategy was divided into two segments: (1) acquisition of patents and (2) acquisition of scientific communications. In both cases, search engines were used, as well as the arrangement of keywords, and Boolean operators were used (cardiac or (medical)) and (simulators or (auscultation)). A 5-year search period was considered, that is, from 2020 to 2024, as the first filter (Filter 1). Subsequently, the collected data were processed for analysis using three filters, which are explained in Section 2.1 and Section 2.2 In addition, duplicate documents were excluded from the dataset. The detailed checklist is available in the Supplementary Materials (Table S1).

### 2.1. Patent Search

**Figure 2:**
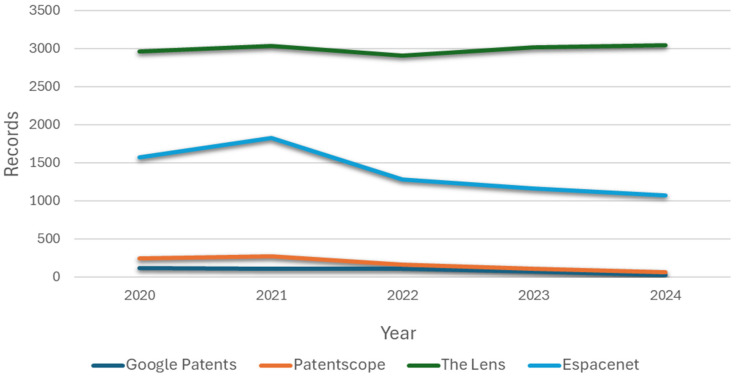
Documents published by year.

Four search engines were used (Google Patents, Patentscope, Espacenet, and The Lens), in which the arrangement of keywords and Boolean operators mentioned in the previous subsection shows a constant in the registration of patents for patient simulators for cardiac auscultation in recent years (see Figure 2).

Subsequently, a filter called Filter 2 related to the international patent classification (IPC) A61B 5/00 and A61B 5/024 was used. These patents belong to the group of simulators specifically designed to teach diagnostic skills, such as auscultation, by simulating heart sounds and other vital signs. This filter was applied directly in the corresponding search engine, since the objective is to determine the prior art of the patient simulator, and this classification ensures relevance by focusing on inventions within the appropriate technological scope.

Filter 3 pertains to including the word “simulator”, which must appear in the patent title. Finally, after obtaining the results from the databases by applying the three filters, a single record was integrated, and “data cleaning” was performed using the free Open Refine^®^ (Version 3.8.7) https://openrefine.org/ (accessed on 17 February 2025). Subsequently, the data were disaggregated, and a manual review of each record was performed, applying criteria for restriction and selection of patents of interest. This action constitutes Filter 4.

Filter 4 was implemented with the aim of ensuring the relevance and timeliness of the patents included in the analysis, focusing exclusively on simulators that integrate cardiac auscultation systems.

The specialized nature of the study required a rigorous filtering of the records identified in the databases, thus enabling a more precise approach. Inclusion criteria were established to identify patents containing the technical features essential to the purpose of the study. Conversely, the exclusion criteria allowed for the removal of records that, although related to medical simulation, did not directly contribute to the specific focus of the study. Taken together, these criteria ensured a rigorous and targeted selection process, thereby enhancing the validity and relevance of the patentometric analysis results.

The following section outlines the inclusion and exclusion criteria applied to the patent selection process.

A. Inclusion criteria:
  - Covers simulators that specifically incorporate a cardiac auscultation system.
  - Describes mechanisms for reproducing normal or pathological heart sounds.
    The patent may be registered in any patent office of any country.
  - Details the design or manufacturing method of simulators with defined anatomical landmarks for auscultation.
  - May be registered in any national or international patent office.
B. Exclusion criteria:
  - Refers to general-purpose simulators without explicit mention of cardiac auscultation.
  - Focused exclusively on other physiological systems.
  - Duplicates across databases—only the first occurrence was retained to avoid redundancy.
  - Filed before 2020, given the typically slow pace of development and dissemination.

Filter 4 consisted of a manual review of the articles that passed the previous stages to verify that they met all the previously established inclusion criteria. This review was carried out independently by two evaluators (C.R.-M. and L.A.Z.-A.). To ensure consistency and standardization of the process, a review guide was developed that included clear operational definitions of each inclusion and exclusion criterion.

During the process, each evaluator independently recorded their decision to include or exclude. In cases where there were discrepancies between the evaluators, a discussion was held to reach a consensus. If agreement could not be reached after the discussion, a third evaluator (J.J.R.-L.) acted as an arbitrator to make the final decision. In order to assess inter-rater reliability, Cohen’s kappa coefficient was calculated. The value obtained was κ = 0.82, indicating a high degree of agreement according to commonly accepted standards. This reliability analysis supports the robustness of the manual selection process and minimizes selection bias.

The discrimination of Filter 4 involved adding all the documents obtained after applying Filter 3. A total of 263 documents were evaluated using the inclusion and exclusion criteria, ultimately identifying 69 documents that contained information about the inventor, registration office, year of publication, patent title, registration key, and international classification of the patent. Table 1 illustrates the effect of reducing records when applying each filter.

### 2.2. Search for Scientific Communications

**Figure 3:**
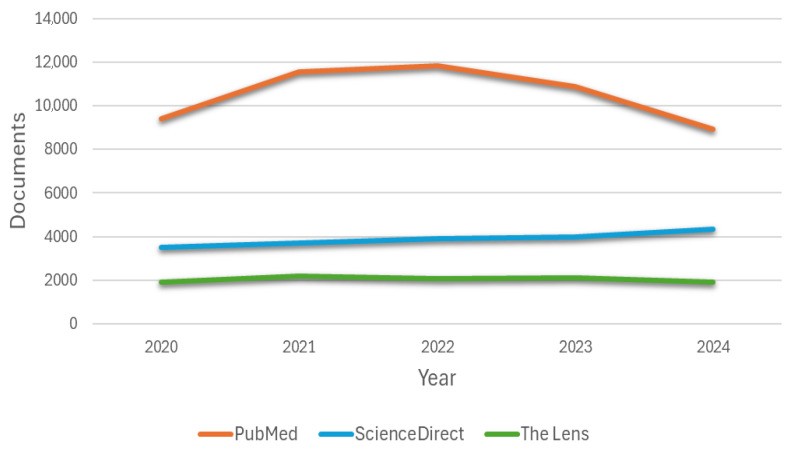
Annual production of articles found related to cardiac simulators used in medical education by three databases.

Three search engines (PubMed, ScienceDirect, and The Lens) were used to analyze the literature, in which the arrangement of keywords and Boolean operators was applied, as well as the period of 5 years as a filter (Filter 1). The number of articles related to cardiac simulators used in medical education per year is shown in Figure 3, where an increasing trend is observed in the first years. In recent years, a decreasing trend has been observed in the case of the PubMed search engine. This contrasts with the patterns seen in ScienceDirect and The Lens, where no clear or consistent trend is evident. The lack of a marked increase or decrease in these platforms may be attributed to their unified systems, which compile articles from broader, indexed databases. Regarding the second filter, Filter 2 is related to including the word “simulator” to more specifically select the relevant articles.

Subsequently, a third filter (Filter 3) was applied to the results from each search engine, using inclusion and exclusion criteria to refine and identify the most relevant information on the topic:

A. Inclusion criteria:
  - Focused on the design of patient simulators for cardiac auscultation.
  - Incorporating clinical cases involving auscultation.
  - Addressing testing and evaluation metrics for cardiac auscultation simulators.
  - Including feedback mechanisms for auscultation techniques.
  - Involving the development of patient simulators for cardiac auscultation.
  - Describing fabrication methods for mannequin simulators.
B. Exclusion Criteria:
  - Generalist simulators without specific reference to cardiac auscultation.
  - Devices are limited to sound recording with no educational component.
  - Simulators dedicated solely to arrhythmia or ECG.
  - Devices focus exclusively on vital sign monitoring.
  - Duplicate records.
  - Items published before 2020.
  - Simulators targeting systems other than the cardiovascular system.

Table 2 shows the effect of reducing records when applying the inclusion and exclusion criteria. A total of 52 records were obtained by performing the bibliometric analysis using free Open Refine^®^ software.

## 3. Results

**Figure 4:**
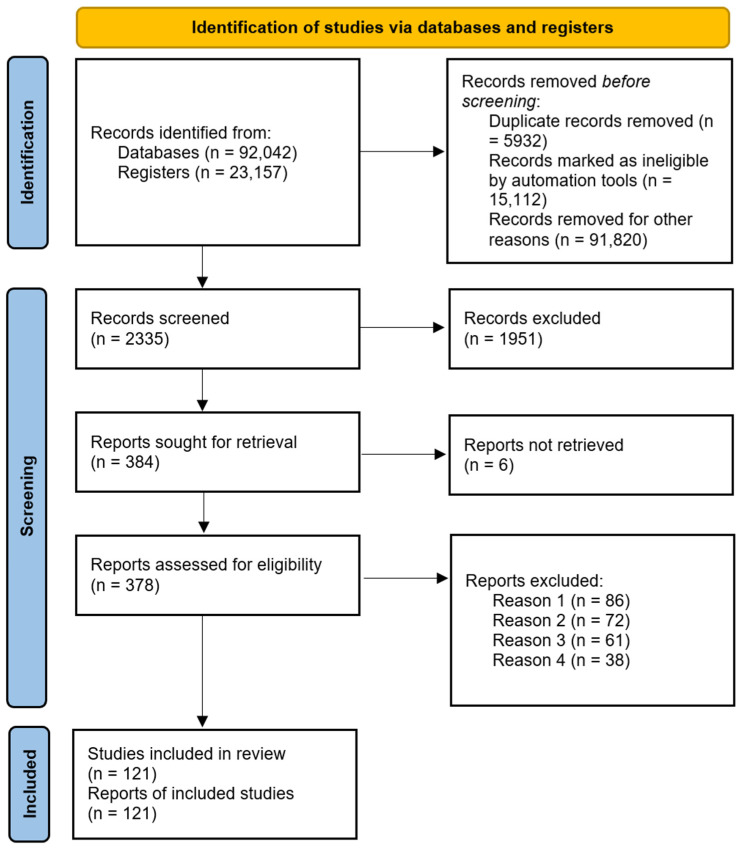
Flowchart corresponding to the Identification of studies via databases and registers.

A total of 115,199 records were found in the database searches. After data cleaning, we examined 2335 records, of which we reviewed 378 full-text papers and finally included 121 [11,12,13,14,15,16,17,18,19,20,21,22,23,24,25,26,27,28,29,30,31,32,33,34,35,36,37,38,39,40,41,42,43,44,45,46,47,48,49,50,51,52,53,54,55,56,57,58,59,60,61,62,63,64,65,66,67,68,69,70,71,72,73,74,75,76,77,78,79,80,81,82,83,84,85,86,87,88,89,90,91,92,93,94,95,96,97,98,99,100,101,102,103,104,105,106,107,108,109,110,111,112,113,114,115,116,117,118,119,120,121,122,123,124,125,126,127,128,129,130,131]. Subsequently, we searched for papers that cited any of the initially included studies, as well as their references. However, no additional articles meeting the criteria were found in these searches (see Figure 4).

### 3.1. Patentometric Analysis

**Figure 5:**
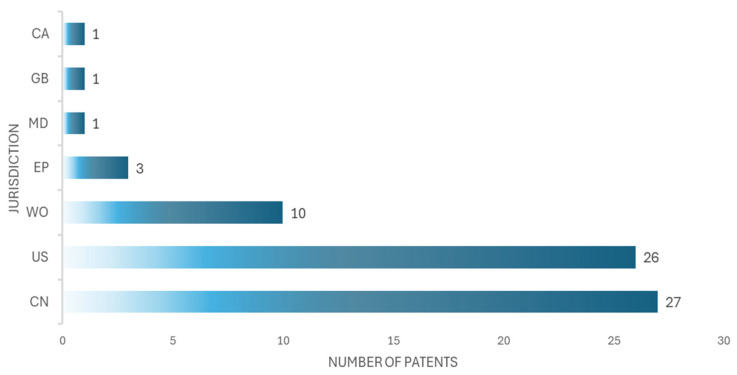
The number of patents in seven jurisdictions is shown, corresponding to China (CN), the United States (US), the Worldwide Office (WO), the European Office (EU), the United Kingdom (GB), Moldova (MD), and Canada (CA).

After filtering the documents, 69 patents related to medical simulation devices were selected; the results are shown in Table 3. The main offices from which the patents were registered are China (CN, 27 patents), the United States (US, 26 patents), the International Office (WO, 10), the European Office (EU, 3 patents), the United Kingdom (GB, 1 patent), Moldova (MD, 1 patent), and Canada (CA, 1 patent). As Figure 5 shows, China has the highest number of patents, although it is not far behind the United States, which has the second highest number of patents.

**Figure 6:**
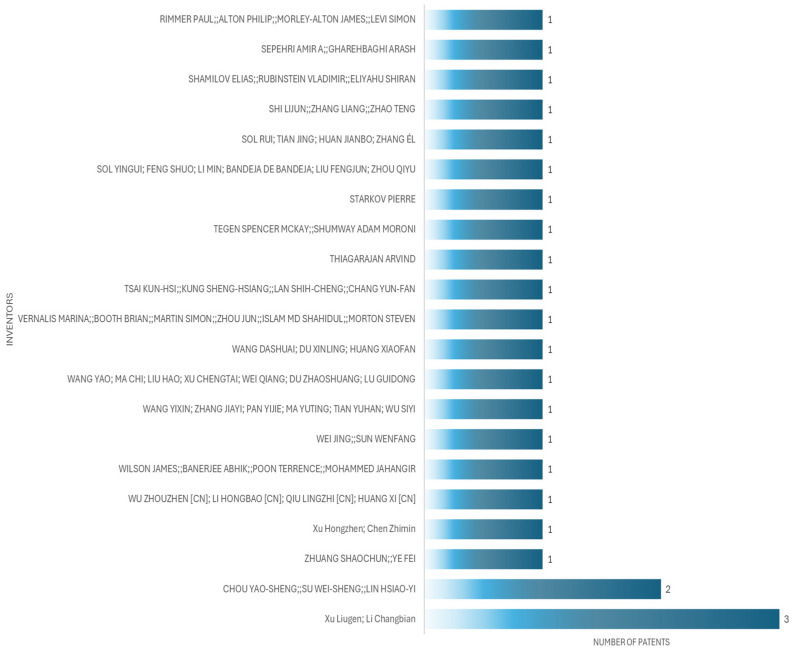
Top patent inventors for patient simulators for cardiac auscultation.

**Figure 7:**
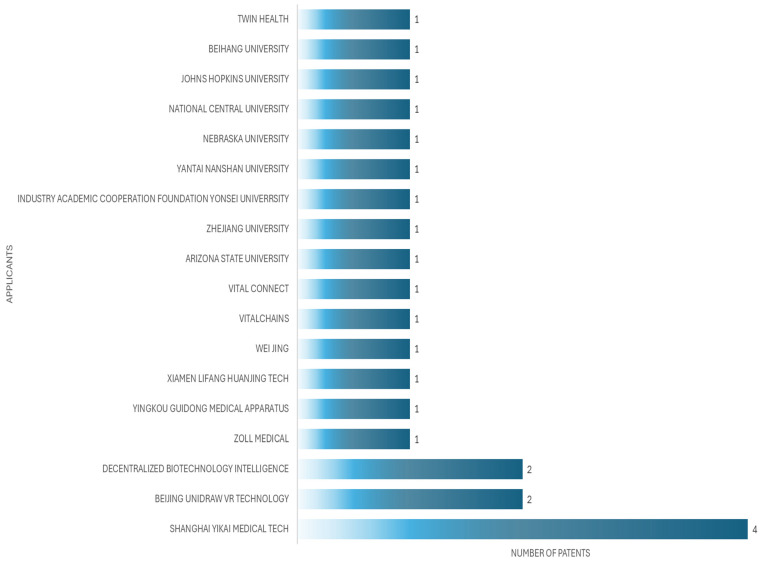
Top patent applicants for patient simulators for cardiac auscultation.

The applicant with the highest number of patents is Xu Liugen, an inventor, who in this case has three patents, from 2020 to 2024. Figure 6 shows the principal inventors of patient simulators for cardiac auscultation, while Figure 7 shows the principal applicants of patient simulators for cardiac auscultation.

**Figure 8:**
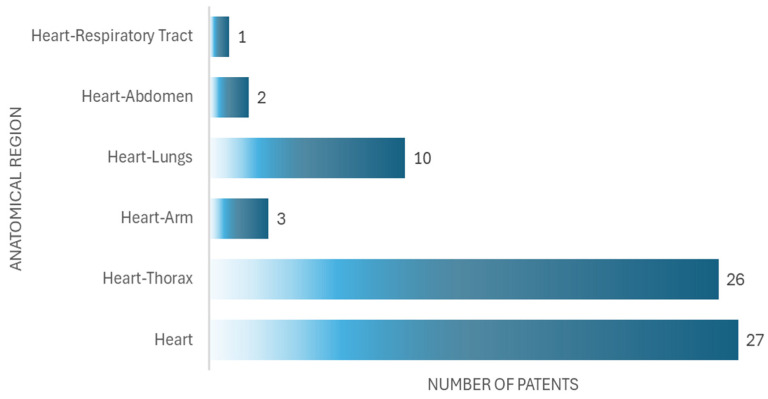
Number of patents by anatomical region.

In the analysis of the patents, we found that 27 documents are related to devices for auscultation exclusively of the heart, and 42 documents are related to devices for auscultation of the heart and another anatomical part, as seen in Figure 8.

**Figure 9:**
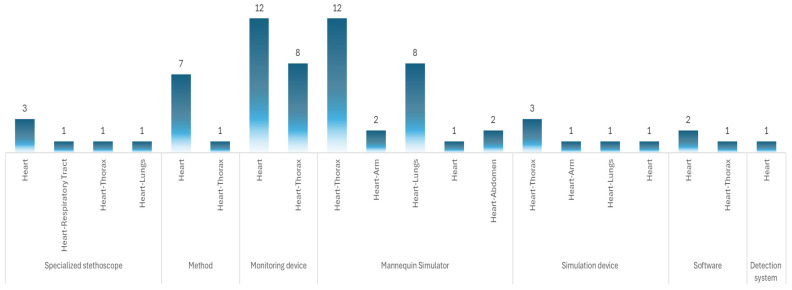
Description of the patents and primary coverage.

Among the 69 results obtained, 25 correspond to “mannequin simulator”, 20 to monitoring device, 6 to specialized stethoscope, 6 to simulation device, 3 to software, and 1 to detection system. In addition, eight results are associated with methodologies, which can be design methods or application methods. Figure 9 shows the resulting patents and the distribution of the body region they represent.

From the patent data, 55 patents refer to patents related only to the thoracic region and physical devices, leaving out methods. In 31 patents, the predominant technology is electronic, using sensors of different characteristics, microprocessors, and controllers as the main elements to simulate cardiac tones. In eight patents, the predominant technology is electromechanical–electronic, using sensors, actuators, and motors with which tones that emulate the heart are made. In seven patents, the technology used is simulation, in which the auscultation process is performed using 3D models, augmented reality, virtual reality, and mixed reality. In four patents, the technology used is artificial intelligence, where, in addition to a physical model, it uses neural networks for continuous learning of the software. In the four patents, the technology used is mechanical, which uses connecting rods, springs, and threaded mechanisms to produce heart tones. For one patent, the technology used is machine learning, supported by a mannequin simulator.

Devices based on electronic technology are not only the most prevalent, 56.3% of the patents analyzed, but also represent a functionally adequate solution for simulating heart sounds.

This technology allows for the easy integration of speakers, sensors, and control systems, facilitating the auditory reproduction of normal and pathological conditions. However, when compared to more advanced technologies, such as artificial intelligence systems, there is a significant difference in terms of educational capabilities: while electronic devices allow for passive and repetitive training, AI-based systems can offer personalized feedback and automatic analysis of user performance. Therefore, although electronics remain the predominant option due to their technical and economic viability, their educational potential is more limited compared to emerging technologies.

**Figure 10:**
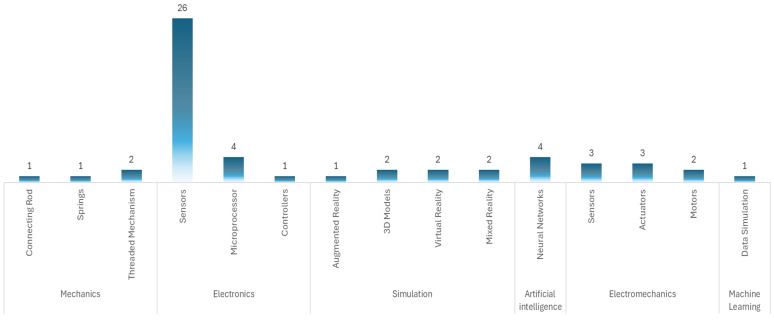
Type of technology used in cardiac simulation.

Table 3 shows the selected patents, the technology used, the type of device, and the anatomical region it simulates. Figure 10 shows the technology used in the patents and the main elements with which they implement the functionality of the simulation device.

### 3.2. Scientometric Analysis

For the articles found in Section 2.2, an analysis was carried out, and the following results were obtained. Specifically, the most recurrent keywords were identified, including: simulation (frequency = 19 articles), training (frequency = 18 articles), simulator (frequency = 17 articles), education (frequency = 17 articles), medical (frequency = 16 articles), virtual reality (frequency = 15 articles), surgery learning (frequency = 11 articles), heart care (frequency = 10 articles), robotic patient (frequency = 6 articles), auscultation (frequency = 3 articles), surgical (frequency = 2 articles), validity (frequency = 2 articles), and assisted (frequency = 1 article).

The keywords identified also allow us to infer relevant aspects of the technological development of the devices. For example, the high frequency of terms such as “virtual reality” and “simulation” suggests a growing incorporation of immersive technologies in the design of medical simulators. The appearance of the term “robotic patient” reflects interest in high-fidelity mannequins with components capable of reproducing physiological functions such as heart sounds, chest movements, or responses to auscultation. Likewise, words such as “training” and “education” indicate that the design of these devices is oriented not only toward anatomical representation but also toward improving clinical skills through repetitive practice and feedback. Therefore, the scientometric trends observed are directly linked to the type of technologies integrated into the simulators and the pedagogical objectives they seek to achieve.

**Figure 11:**
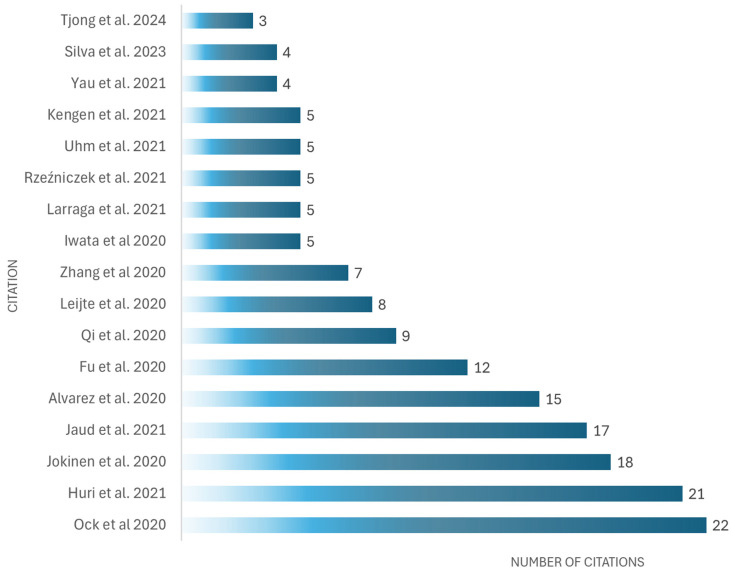
Most cited documents related to cardiac simulators used in medical education, as cited in The Lens and Google Scholar, February 2025 [83,84,85,93,94,97,98,105,107,108,115,118,121,124,126,128,130].

Regarding the authors, it was found that some have the same publications: Yutaka Kagaya, Leijte E., Pinto L., Fu Y., and Qi Z.; however, Ock J. is the most cited author in this field related to cardiac simulators used in medical education (see Table 4 and Figure 11).

**Figure 12:**
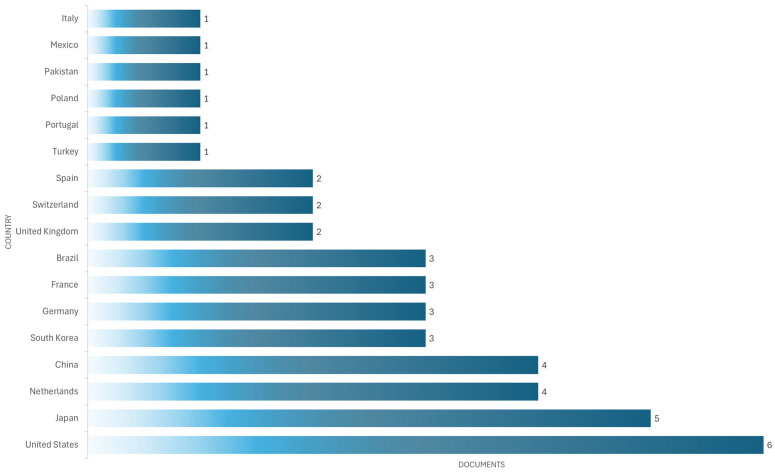
The most productive countries in developing simulator devices for cardiac auscultation.

The United States is the most productive country (six documents), followed by the Japan (five documents), China (four documents), and the Netherlands (four documents) (see Figure 12).

As shown in Figure 12, most studies come from high-income countries such as the United States, Japan, and the Netherlands, suggesting greater adoption and investment in these technologies. In contrast, middle- and low-income countries such as Mexico, Pakistan, and Turkey have a much smaller share, reflected in limited output with only one document per country. This disparity can be attributed to multiple barriers: the high initial investment required to acquire high-fidelity simulators, limited technological infrastructure, and a lack of specialized training programs. Furthermore, in some contexts, medical curricula do not yet systematically integrate simulation as a pedagogical strategy. In contrast, institutions in countries with greater resources tend to have fully equipped simulation laboratories and educational policies that promote their use. These regional differences must be considered when designing global medical education policies, promoting strategies for collaboration, technology transfer, and the development of low-cost simulators for contexts with limited resources.

Table 5 shows the selected articles used in the scientometric analysis employed, database results, and selection criteria. The presentation of the performance parameters of each selected article is essential, as it allows us to identify the criteria used to evaluate the effectiveness of medical simulators in educational and clinical settings. This information not only facilitates comparison between different types of simulators, it also helps to detect trends and limitations in the validation methods applied.

## 4. Discussion

In this review of 69 patents and 52 scientific articles, different analyses have been used to find similarities to guide new proposals that may arise in future designs. It was concluded that auscultation simulators have advanced from basic mechanical models to electronic devices with digitized sounds and artificial intelligence systems that allow the simulation of different pathologies. However, limitations were identified in the clinical validation of some commercial devices, suggesting the need for a greater correlation between patented developments and their implementation in educational and hospital environments.

The review shows an 8% increase in the implementation of devices used for cardiac auscultation with artificial intelligence (AI) compared to traditional models (see Figure 10). This trend suggests a move toward the integration of more sophisticated technologies, which can improve diagnostic accuracy and the detection capacity of a wide range of cardiac pathologies.

On the other hand, the patents reviewed show an emphasis on the integration of new technologies, such as different sensors, wireless connectivity, and augmented reality. However, many of these innovations have not been extensively evaluated in academic studies, which generates uncertainty about their real impact on the practical teaching of auscultation. In addition, it is noted that several patents focus on technical improvements without addressing key aspects such as affordability and compatibility with stethoscopes used in clinical practice. Another important finding is the gap between technological innovation and its adoption in medical training. Despite the increasing sophistication of simulators, their availability in teaching institutions remains limited, mainly due to production and acquisition costs. In addition, 85% of the studies analyzed reported ≥90% compliance with ISO 13485:2016. Medical devices–Quality management systems–Requirements for regulatory purposes. International Organization for Standardization (ISO): Geneva, Switzerland, 2016. reflecting strong alignment with international regulatory standards for medical devices (see Table 3).

If the simulator complies with ISO 13485, it can be guaranteed that heart sounds are reproduced accurately and at the correct points. This improves auditory and anatomical learning, which are key to clinical auscultation, but it does not assess whether students are learning, only that the simulator is well designed, safe, and functions as it should.

This suggests that future research should focus on the development of low-cost devices, taking advantage of technologies such as 3D printing and open-source software.

Although technological advances in auscultation simulators have been made, one of the main barriers to their adoption in medical training is the transition of these devices from the academic to the clinical setting. While current simulators have proven to be effective in controlled training scenarios, their integration in hospitals and clinics remains limited due to several factors, such as production cost, lack of trained personnel to operate them, and the difficulty of integrating these technologies into existing clinical systems.

Currently, many devices with integrated artificial intelligence are in the early stages of development or validation. Although some have proven effective in controlled environments, their rigorous clinical validation is still limited and poses a challenge, especially given the need for multicenter studies with large samples and standardized protocols. In addition, regulatory criteria vary between countries, complicating their widespread clinical adoption.

It is critical to address these challenges through the creation of simulators that are more accessible and easier to implement in the hospital setting. One possible solution would be the development of simulators that can be directly integrated with hospital patient management systems, allowing for better teaching and real-time assessment of auscultation skills. This would not only improve the quality of training but would also contribute to better preparation of healthcare professionals when facing real clinical situations. The effectiveness of the simulator often depends on the human instructor guiding the session. Without an appropriate teaching strategy, even high-fidelity simulators can lead to poor or incorrect learning.

The predominance of multiregional simulators could suggest an advantage in terms of educational versatility. However, our analysis suggests that this breadth of coverage may be accompanied by less specialization in the reproduction of heart sounds. In contrast, simulators designed exclusively for cardiac auscultation tend to have superior acoustic fidelity and a deeper focus on auscultation points, which may specifically benefit cardiology training.

One area that could significantly benefit the development of auscultation simulators is the active involvement of end users in the design process. Involving physicians, medical students, nurses, and other healthcare professionals in the design and testing phases of simulators allows direct feedback on the usability, educational effectiveness, and areas for improvement of the devices. This collaborative approach helps identify practical issues that may not be evident in the initial design, such as the ergonomics of the simulator or the clarity of the user interfaces.

### 4.1. Structural Elements of the Simulation Mannequin

**Figure 13:**
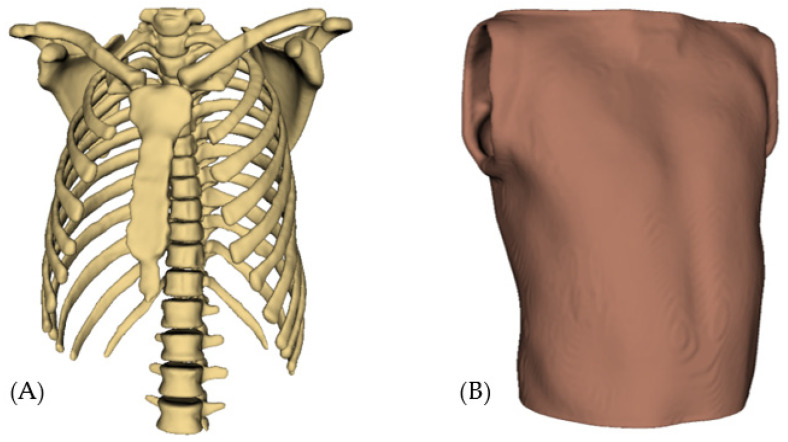
Basic structure of the mannequin simulator: (**A**) Thorax; (**B**) Skin.

According to the selected patents and scientific documentation, the design of the patient simulator mannequins consists of a realistic anatomical torso, which must accurately represent the human thoracic anatomy, including anatomical references such as the sternum, ribs and intercostal spaces. It should also have well-defined auscultation points to ensure proper practice (see Figure 13).

### 4.2. Types of Materials

In the patents reviewed, various types of materials were identified as commonly used in medical simulation mannequins. For skin and soft tissues, materials such as medical-grade silicone are employed due to their flexibility and texture, which closely resemble human skin. This provides greater elasticity and a more realistic tactile experience. Such properties are crucial in medical training devices, as they can enhance the transmission of sound through the mannequin’s surface.

As for the internal structure and support, rigid plastics are commonly used because of their strength and ease of molding. Notably, at least 36% of the devices examined in the patent review incorporate both medical-grade silicone and rigid plastics, highlighting their widespread adoption in the design and manufacturing of medical simulators (see Figure 9).

**Figure 14:**
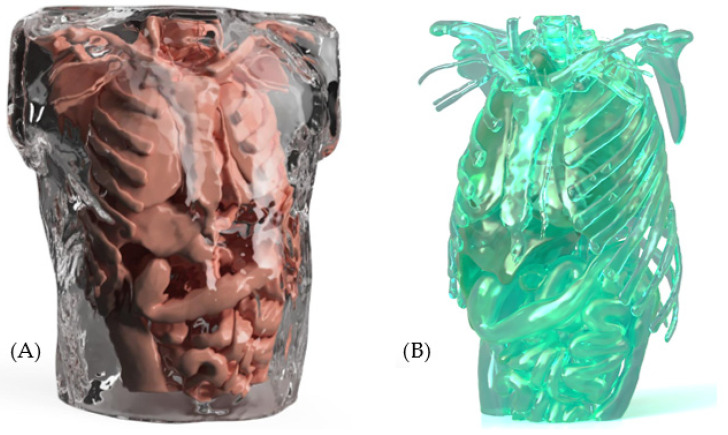
Type of material: (**A**) External structure; (**B**) Internal structure.

To illustrate the aforementioned material applications, two renderings generated from a proprietary design proposal are presented. Image (A) shows the external surface, which simulates skin and soft tissues using medical-grade silicone, highlighting its realistic texture, transparency, and flexibility. These visual renderings serve to exemplify how the selected materials contribute to the functionality and realism of the medical simulation mannequin. Image (B) shows the internal structure of the mannequin, highlighting the use of rigid plastics to provide structural support and maintain anatomical integrity (see Figure 14).

### 4.3. Types of Materials for Sound Simulation

**Figure 15:**
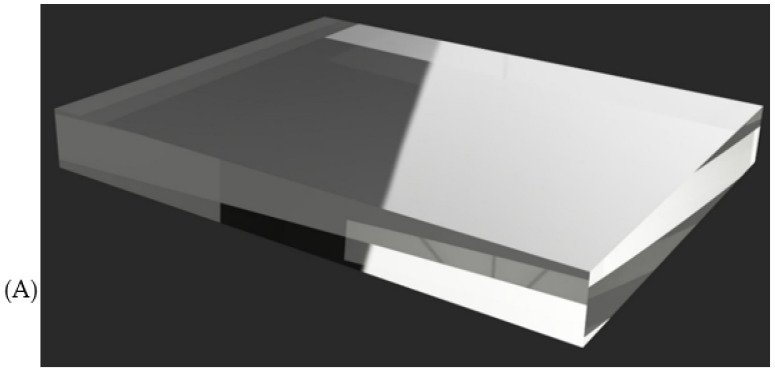

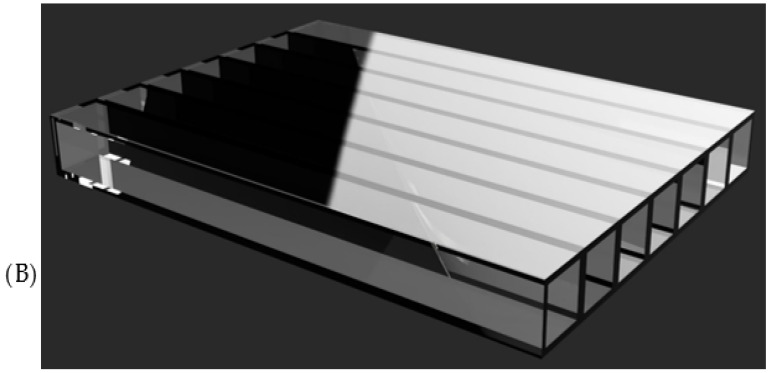
Types of resonance chamber material used: (**A**) acrylic; (**B**) polycarbonate.

For sound distribution and amplification, acrylic or polycarbonate plates are used to function as resonance chambers, allowing vibrations to propagate uniformly inside the mannequin. These materials help to avoid distortions and improve sound perception at the correct auscultation points. In addition, some simulator models incorporate silicone gel or acoustic foams to control sound absorption and reduce unwanted interference within the mannequin structure. These materials allow the clarity and direction of sound to be adjusted to make the user experience as realistic as possible (see Figure 15).

From the patents and research articles, it was determined that the choice between acrylic and polycarbonate depends on factors such as mechanical strength and sound transmission quality. While acrylic offers greater rigidity and clarity in the propagation of vibrations, polycarbonate stands out for its high impact resistance and flexibility, making it ideal for more resistant or complex-shaped structures.

In addition, the mannequin design presented in this work has been the subject of an industrial design registration application, specifically the industrial model, which supports the originality and innovation of the proposal. The images included in this article show some of the key technical features of the mannequin, such as the anatomical arrangement. These features were considered in the application with application number MX/f/2025/001145, currently pending before the Mexican Institute of Industrial Property (IMPI). The legal protection seeks to guarantee the authorship of the design and to promote its use in educational contexts, particularly in medical training.

## 5. Conclusions

Cardiac auscultation simulator mannequins represent an essential tool in medical education, allowing students to develop clinical skills in a controlled and safe environment. Through this review, it has become evident how the combination of acoustic materials, simulation technologies, and ergonomic designs has significantly improved the fidelity and effectiveness of these devices. The results highlight the evolution of these simulators, from basic mechanical models to advanced systems that integrate acoustic optimization materials, electronic feedback, and remote control capabilities.

The review found an 8% increase in the adoption of AI auscultation devices, reflecting a trend toward more accurate and automated technologies. Furthermore, 85% of studies reported ≥90% compliance with ISO 13485, highlighting the priority placed on simulator safety and quality. These advances suggest significant potential for improving diagnostic accuracy and medical education. However, further clinical validation is required to ensure their effectiveness in real-world settings.

Of the 69 patents analyzed, 27 (39.1%) focus exclusively on cardiac auscultation, while 42 (60.9%) cover multiple anatomical regions. When analyzing the citations received and the reported use in educational validation studies, it is observed that multiregional simulators tend to be cited more frequently in studies that integrate comprehensive clinical training. However, cardiac-auscultation-specific simulators show a higher level of detail in their acoustic design and are preferred in research focused on in-depth auscultation skills.

Although the principles behind the technologies remain unchanged, some devices incorporate various technologies and materials to offer a realistic learning experience. They use sound systems with speakers or transducers to reproduce accurate heart tones, along with acoustic materials such as acrylic, polycarbonate, and silicone gel to optimize sound propagation. In addition, many models are compatible with conventional stethoscopes or include electronic versions with simulated pathology settings. Remote connectivity allows instructors to modify parameters in real time, while some advanced devices offer automatic feedback.

Based on the findings, several promising lines of research are identified to strengthen the development of medical simulators. First, it is recommended to promote the integration of artificial intelligence (AI) with real-time feedback systems, which would allow for dynamic adaptation of the simulator to user responses and automated performance evaluation. In addition, there is a clear need to move toward the standardization of validation protocols, which would allow for the comparison of educational effectiveness between different simulators under common and reproducible criteria. Finally, given the growing importance of remote medical care, it is suggested that the use of simulators in telemedicine training contexts be explored, particularly for training clinical skills through virtual platforms that simulate real remote consultation scenarios.

The contribution of these results focuses on the technologies and techniques employed and the accessibility and standardization of these simulators, which opens the door to future research and improvements in their development. In addition, this work contributes to the formulation of the design presented in Section 4.1, Section 4.2 and Section 4.3 as a prototype of a patient simulator mannequin for the practice of cardiac auscultation.

## Figures and Tables

**Table 1:** Results of the patent search across various search engines.

Search Engines	Filter 1	Filter 2	Filter 3	Filter 4
Patentscope	836	31	16	69
Google Patents	432	27	21	
Espacenet	6918	220	53	
The Lens	14,971	1951	182	
Total	23,157	2229	272	69

**Table 2:** Total number of documents collected from search engine queries.

Search Engines	Filter 1	Filter 2	Filter 3
The Lens	11,680	19	52
ScienceDirect	22,460	34	
PubMed	59,902	53	
Total	92,042	106	52

**Table 3:** Cardiac simulation device patents.

Cite	Title	Main Applicant	Body Part	Type	Technology
[12]	Auscultation Device for Cardiovascular Medicine Department	Wei Jing	Heart	Specialized stethoscope	Mechanics
[13]	Auscultation Position Indication Method and Device	Huawei Tech	Heart	Method	Electronics
[14]	Auscultation System	Ausculsciences	Heart	Method	Electronics
[15]	Auscultation System for Guiding a User to Perform Auscultation on a Subject	Aria Narayan Vikram	Heart	Monitoring device	Simulation
[16]	Auscultation Training Examination Model	Zhejiang University	Heart–Lungs	Mannequin simulator	Electromechanics
[17]	Biofeedback Apparatus and Method	Gideon Eric	Heart–Respiratory Tract	Specialized stethoscope	Mechanics
[18]	Biosignal Measurement Apparatus and Method	Myllylae Teemu	Heart	Monitoring device	Electronics
[19]	Cardiac Health Assessment Systems and Methods	Cortery Ab	Heart	Monitoring device	Electronics
[20]	Cardiopulmonary Auscultation Virtual Simulation Training System and Method	Beijing Unidraw VR Technology	Heart–Lungs	Simulation device	Simulation
[21]	Cardiopulmonary Percussion Model for Teaching	Union Hospital Tongji Medical College Huazhong University of Science and Technology	Heart–Lungs	Mannequin simulator	Electromechanics
[22]	Cardiopulmonary Simultaneous Multidirectional Auscultation Anthropomorphic Dummy	Shanghai Yikai Medical Tech	Heart–Lungs	Mannequin simulator	Mechanics
[23]	Chest Wall Oscillation System with Digital Auscultation	Koninklijke Philips	Heart–Thorax	Monitoring device	Electromechanics
[24]	Comprehensive Delivery Mother-Child First-Aid Model	Xi’an Mcneill Medical Technology	Heart–Thorax	Mannequin simulator	Electronics
[25]	Comprehensive Skill Training Simulator	Shanghai Qize Medical	Heart–Thorax	Mannequin simulator	Electronics
[26]	Device for Automated Screening of Congenital Heart Diseases	Capis Sprl	Heart	Monitoring device	Artificial intelligence
[27]	Diagnostic Simulator	Seno Medical Instr	Heart–Thorax	Monitoring device	Electronics
[28]	Digital Stethoscope for Diagnosing Cardiopulmonary Pathologies	Alsaliem Sulaiman	Heart–Lungs	Specialized stethoscope	Electronics
[29]	Dynamic Body Part Simulator	Bioconix Pty	Heart–Thorax	Mannequin simulator	Electromechanics
[30]	Electronic Device for Auscultation and Method of Operation Thereof	Smartsound	Heart	Specialized stethoscope	Artificial intelligence
[31]	Electronic Standardized Human Body Model System for Emergency Training and Examination	Beijing Shengyi Zhijiao Technology	Heart–Arm	Mannequin simulator	Electronics
[32]	Electronic Standardized Infant Model System for Emergency Training and Examination	Guangdong Medical Technology	Heart–Thorax	Mannequin simulator	Electronics
[33]	First-Aid Skill Training Model with Airway Management Function	Second Military Medical University	Heart–Thorax	Mannequin simulator	Electronics
[34]	Heart Sound Deep Learning Heart Disease Prediction Method and System for Auscultation Teaching	The Sixth Medical Center of Chinese Pla General Hospital	Heart	Software	Artificial intelligence
[35]	Heart-Variable Simulation Device for Cardiopulmonary Auscultation Palpation	Shanghai Yikai Medical Technology	Heart–Lungs	Mannequin simulator	Electromechanics
[36]	High-Simulation Multifunctional Wireless Human Body Vital Sign Examination Training Model	Yingkou Guidong medical apparatus	Heart–Lungs	Mannequin simulator	Electronics
[37]	Human Pulse and Heartbeat Simulator	Laerdal Medical	Heart	Simulation device	Electronics
[38]	Integrated Sensing Device for Heart Sound and ECG Signals	Decentralized Biotechnology Intelligence	Heart	Monitoring device	Electronics
[39]	Modeling Method, Apparatus, Device, and Storage Medium of Dynamic Cardiovascular System	Beihang University	Heart	Method	Simulation
[40]	Multi-Mic Sound Collector and System and Method for Sound Localization	Vitalchains	Heart–Thorax	Monitoring device	Electronics
[41]	Multi-Modal Heart Diagnostic System and Method	University of Alabama	Heart	Monitoring device	Electronics
[42]	Multi-Parameter Simulator	Contec Medical Systems Qinhuangdao	Heart–Thorax	Simulation device	Electronics
[43]	Noninvasive Arterial Condition Detecting Method, System and Non-Transitory Computer Readable Storage Medium	National Central University	Heart	Detection system	Electronics
[44]	Portable Heart Motion Monitor	Cardiac Motion; University of California	Heart	Monitoring device	Electronics
[45]	PPG Pulse Wave Simulator	Hunan Cofoe Xinchi Medical Tech	Heart–Arm	Simulation device	Electronics
[46]	Pregnant Woman four-step Palpation Simulation Teaching Model	Beijing Yimo Technology	Heart–Thorax	Mannequin simulator	Electronics
[47]	Pulse Simulator and Simulation Method Thereof	Yantai Nanshan University	Heart–Thorax	Simulation device	Electronics
[48]	Pulse Wave Conduction Parameter Measuring Method and Pulse Wave Conduction Parameter Processing Device	Shenzhen Darma Tech	Heart	Monitoring device	Electronics
[49]	Real-Time Adaptation of a Personalized Heart Model	Sorin Crm	Heart	Method	Electromechanics
[50]	Screening Device, Method and System for Structural Heart Disease	Vital Connect	Heart	Method	Electronics
[51]	Simulating Clinical Trials Using Whole Body Digital Twin Technology	Twin Health	Heart–Thorax	Simulation device	Simulation
[52]	Simulation Defibrillator Teaching Equipment with Cardio-Pulmonary Resuscitation Display Function	Guangdong Kangwei Technology	Heart–Thorax	Mannequin simulator	Electronics
[53]	Simulation Device for Palpation of Variable Lungs Through Cardiopulmonary Auscultation	Shanghai Yikai Medical Tech	Heart–Lungs	Mannequin simulator	Mechanics
[54]	Simulation Doll	ExAc	Heart	Mannequin simulator	Simulation
[55]	Simulation of Heart Pacing for Modeling Arrhythmia	Biosense Webster Israel	Heart	Method	Simulation
[56]	Simulation System for Abdominal Cavity Examination	Obshchestvo S Ogranichennoy Otvetstvennostyu Medviar	Heart–Abdomen	Mannequin simulator	Electromechanics
[57]	Stethographic Device	Western Michigan University	Heart–Thorax	Monitoring device	Electronics
[58]	Stethoscope Dummy	Qi Jingtan	Heart–Lungs	Mannequin simulator	Electronics
[59]	System and Method for Determining An Auscultation Quality Metric	Johns Hopkins University	Heart–Thorax	Method	Artificial intelligence
[60]	System and Method for Evaluating Effects of Antiarrhythmic Agent	Industry-Academic Cooperation Foundation Yonsei University	Heart	Software	Machine learning
[61]	System And Method for Medical Simulation	Global Diagnostic Imaging Solutions	Heart–Thorax	Software	Simulation
[62]	System For Recording Chest Signals and Method Using Said System	CSEM	Heart–Thorax	Monitoring device	Electronics
[63]	System, Device and Method for Automated Auscultation	Andino Jean	Heart–Thorax	Monitoring device	Artificial intelligence
[64]	System, Device and Method for Automated Auscultation	Arizona State University	Heart–Thorax	Monitoring device	Electronics
[65]	Systems and Methods for Determination of Pulse Arrival Time with Wearable Electronic Devices	Nebraska University	Heart	Monitoring device	Electronics
[66]	Systems and Methods for Determining Filtered Cardiac Output	Edwards Lifesciences	Heart	Monitoring device	Electronics
[67]	Systems and Methods for Measuring Patient Vital Signs	Thiagarajan Arvind	Heart–Thorax	Specialized stethoscope	Electronics
[68]	Systems and Methods for Testing a Medical Device	Zoll Medical	Heart–Thorax	Mannequin simulator	Electromechanics
[69]	Systems, Devices, and/or Methods for Managing Health	Tegen Spencer Mckay	Heart	Monitoring device	Electronics
[70]	Teaching Device for Learning Four Palpation and Fetal Heart Auscultation	Xiamen Lifang Huanjing Tech	Heart–Thorax	Mannequin simulator	Electronics
[71]	Teaching is with Simulating People’s Belly Palpation Analogue Means	Shanghai Yikai Medical Tech	Heart–Abdomen	Mannequin simulator	Electronics
[72]	Trachea Cannula Simulation Training Method and System	Beijing Unidraw VR Technology	Heart–Thorax	Mannequin simulator	Simulation
[73]	Training Anthropomorphic Dummy of Hypothermia War Wound	General Hospital of Shenyang Military Region	Heart–Thorax	Mannequin simulator	Electronics
[74]	Training Manikins	Simcraft Tech	Heart	Method	Simulation
[75]	Vein Simulator System	Becton Dickinson	Heart–Arm	Mannequin simulator	Electronics
[76]	Vital Sign Comprehensive Simulator Convenient to Install	Shenzhen Kwda	Heart–Thorax	Mannequin simulator	Mechanics
[77]	Vital Sign Measurement Device	AMI Industries	Heart–Thorax	Monitoring device	Electronics
[78]	Wearable Auscultation Device	Senti Tech^®^	Heart	Monitoring device	Electronics
[79]	Wearable Heart Sound Detection System and Method Thereof	Decentralized Biotechnology Intelligence	Heart	Specialized stethoscope	Electronics
[80]	Wireless Intelligent Cardiopulmonary Auscultation Anthropomorphic Dummy	Henan Yujing Technology Development	Heart–Lungs	Mannequin simulator	Electronics

**Table 4:** Publication trends of leading authors across the years.

Author	Year				
	2020	2021	2022	2023	2024
Yutaka Kagaya		1	1		
Leijte E.	1	1			
Pinto L.					2
Fu Y.	1				1
Qi Z.	1				1
Iwata M.	1				
Rzeźniczek P.	1				
Kengen B.	1				
Ock J.		2			1

**Table 5:** Articles analyzed about simulator devices for cardiac auscultation.

Cite	Main Author	Performance Parameters	Year
[81]	Bai F	Accuracy of volume measurements with both methods and reproducibility of measurements.	2024
[82]	Manzanares A	Learning time to master basic simulated navigation skills and ability to transfer skills from the simulator to real situations.	2024
[83]	Leijte E	Time to complete tasks, economy of motion, and appearance validity of the robotic simulator for advanced suturing tasks.	2020
[84]	Huri G	Accuracy in the performance of arthroscopic procedures and time required to complete standardized surgical tasks.	2021
[85]	Fu Y	Task completion time, use of the CUSUM method to analyze intracorporeal enhancement and errors made.	2020
[86]	Massone C	Detailed analysis of the features that simulate Spark Nevus and their importance in differentiating between lesions.	2023
[87]	Geissler ME	Object transfer, circular cut, balloon resection, and suture, measuring execution time and cognitive load using the NASA-TLX scale.	2024
[88]	Lee JE	Medical students’ perceptions of patient safety before and after simulator training.	2024
[89]	Suzuki H	Evaluation of ease of application and quantity used in a simulated 10.1093/europace/euae037ment.	2022
[90]	Hendrickx DM	Evaluation of the accuracy of simulations and their usefulness in planning public health interventions.	2021
[91]	Yoshimura T	Evaluation of anatomical accuracy by comparison of actual and modeled coordinates.	2021
[92]	Bhattacharya S	Analysis of the relationship between demographic characteristics, driving history, and visuo-cognitive test results with driving simulator performance.	2023
[93]	Silva VC	Assessment of motor, cognitive, and visual functions in older drivers using a simulator.	2023
[94]	Larraga-García B	Evaluation of training efficacy and differences in treatment approaches between medical students and physicians.	2021
[95]	Ulises Sánchez-Vásquez	Ability of the simulator to reproduce normal heart sounds and evaluation of the fidelity of the simulator compared to actual clinical practice.	2023
[96]	Fu TT	Effectiveness of the virtual simulation teaching system in neonatal PICC care training.	2024
[97]	Iwata M	Evaluation of simulator sensitivity in different driving scenarios and applicability of the simulator in clinical studies.	2020
[98]	Yau SY	Comparison of performance between different levels of medical experience and evaluation of applied force during difficult scenarios.	2021
[99]	Kliem PSC	Duration of evaluation and improvement with clinical experience and confidence of participants in their evaluations.	2024
[100]	Yutaka Kagaya	Diagnostic accuracy in the identification of heart murmurs (aortic and mitral regurgitation) and correlation with student satisfaction.	2021
[101]	Hilleke S	The combination of simulator training followed by clinical observation.	2024
[102]	Scott-Watson M	Improvement in basic arthroscopic skills and correlation with personal characteristics.	2024
[103]	von Bernstorff M	A moderate correlation was found between the simulator and a reaction timer, suggesting that simpler tools could estimate BRT in clinical practice.	2021
[104]	Maita H	The use of the bladder simulator significantly improved students’ skills and confidence.	2023
[105]	Rzeźniczek P	Symptoms of simulator sickness measured with the Simulator Sickness Questionnaire (SSQ) before and after simulation.	2020
[106]	Liu C	Effectiveness in background exam training and willingness to use the simulator in the future.	2024
[107]	Jaud C	Benchmark for competency-based training, effectively differentiating between novice and experienced surgeons.	2021
[108]	Kengen B	Task duration between high- and low-impulsivity groups and number of errors made.	2020
[109]	Yutaka Kagaya	Accuracy in identifying heart sounds, student satisfaction, and progress in auscultation skills.	2022
[110]	Pinto L	Success rate between groups before and after simulator training.	2024
[111]	Sønderup M	Accuracy of image placement, number of repetitions required to complete the task, and comparison between novice and experienced surgeons.	2024
[112]	Ghazanfar S	Performance in robotic simulator tasks and transfer of skills to robotic surgery.	2021
[113]	Ducloyer JB	Task execution time and incidence of complications, such as rupture of the posterior capsule.	2024
[114]	Ono Y	Lowest peak forces applied to structures during direct and indirect practice in novices.	2020
[115]	Ock J	Realism of the simulation, ease of use of the simulator, and applicability in the training of medical doctors.	2020
[116]	Motov S	Performance on simulator tasks, progress in technical skills, and participant satisfaction.	2024
[117]	Grethlein D	Correlation with test performance.	2020
[118]	Jokinen E	Comparison between simulator-trained and non-simulator-trained residents.	2020
[119]	Weimer JM	Accuracy in identifying cardiac structures, transfer of simulator skills to real patients, and time required to complete examinations.	2025
[120]	Pinto LOAD	Procedure time, instrument manipulation accuracy, and success rate in simulated procedures.	2024
[121]	Qi D	Accuracy in the execution of the procedure and errors made during the simulation.	2020
[122]	Huang C	Ability to make clinical decisions in emergency situations, application of knowledge in simulated scenarios, and satisfaction and confidence of the participants.	2020
[123]	Gulbakit Koshmaganbetova	Accuracy in identifying heart murmurs, improvement in auscultation skills after training, and comparison between different training durations.	2021
[124]	Zhang CT	Reaction time, rate of safe responses, and impact of variables such as age and distraction.	2020
[125]	Qi Z	Accuracy of navigation, ease of use of the system, and cost and accessibility of the system.	2024
[126]	Uhm D	Gripping technology and sealing forces.	2021
[127]	Leijte E	Time to complete basic tasks, accuracy and efficiency of instrument manipulation, and comparison between different levels of experience of participants.	2021
[128]	Alvarez-Lopez F	Depth perception, ease of use, relevance as a learning tool, and feedback provided by the simulator.	2020
[129]	Porto JT	Coordination, instrument navigation, and timing of procedures.	2020
[130]	Tjong FVY	Percentage of respondents who believe that simulators should be part of routine training and proportion of respondents who suggest that simulation programs should be developed by EHRA.	2024
[131]	Bogar PZ	Exercise evaluation time and efficacy and reliability of the artificial-intelligence-based evaluation approach compared to the standard human-based method.	2024
[132]	Yaïci R	Content validity, construct validity, and criterion validity.	2024

## Supplementary Materials

The following supporting information can be downloaded at: https://www.mdpi.com/article/10.3390/bioengineering12070731/s1, Table S1: Prisma 2020 Checklist.
